# Anthropogenic refugia ameliorate the severe climate-related decline of a montane mammal along its trailing edge

**DOI:** 10.1098/rspb.2012.1301

**Published:** 2012-08-15

**Authors:** Toni Lyn Morelli, Adam B. Smith, Christina R. Kastely, Ilaria Mastroserio, Craig Moritz, Steven R. Beissinger

**Affiliations:** 1 Museum of Vertebrate Zoology, University of California, 3101 Valley Life Sciences, Berkeley, CA 94720, USA; 2 Department of Environmental Science, Policy and Management, University of California, 130 Mulford Hall, Berkeley, CA 94720, USA; 3 Center for Conservation and Sustainable Development, Missouri Botanical Garden, 4344 Shaw Boulevard, Saint Louis, MO 63110, USA

**Keywords:** climate change, ground squirrel, range shift, refugia, Sierra Nevada, trailing edge

## Abstract

We conducted detailed resurveys of a montane mammal, *Urocitellus beldingi*, to examine the effects of climate change on persistence along the trailing edge of its range. Of 74 California sites where *U. beldingi* were historically recorded (1902–1966), 42 per cent were extirpated, with no evidence for colonization of previously unoccupied sites. Increases in both precipitation and temperature predicted site extirpations, potentially owing to snowcover loss. Surprisingly, human land-use change buffered climate change impacts, leading to increased persistence and abundance. Excluding human-modified sites, *U. beldingi* has shown an upslope range retraction of 255 m. Generalized additive models of past distribution were predictive of modern range contractions (AUC = 0.76) and projected extreme reductions (52% and 99%, respectively) of *U. beldingi's* southwestern range to 2080 climates (Hadley and CCCMA A2). Our study suggests the strong impacts of climate change on montane species at their trailing edge and how anthropogenic refugia may mitigate these effects.

## 1. Introduction

Populations can respond to ongoing and accelerating climate change through migration, adaptation or local extinction. Climate change may especially threaten populations at their equatorial or lower margins [1]; their lower adaptive capacity and reduced fitness may make trailing edge populations more likely to go extinct than to adapt [2,3]. Thus, the trailing edge can be particularly informative in understanding how species and ecosystems are responding to increasing temperatures and shifting precipitation regimes. Yet, evidence for retractions at trailing range edges is limited [4,5], possibly as a result of lag effects in climate change responses [6] or of climate change refugia [7,8]. Detailed, long-term data are needed to disentangle the effects of climate change from other causes of range change that affect trailing edge populations [9,10] as well as to provide information to inform predictions of climate change impacts.

Mountain systems harbour exceptional and particularly vulnerable biodiversity [11]; montane mammals may be especially prone to range shifts or population extirpations in response to recent climate change. Most montane mammals have restricted dispersal ability and limited dispersal options [12,13]. Many montane mammals are habitat or dietary specialists and, as they usually experience more extreme seasonality, are especially vulnerable to phenology shifts, resulting in mismatches between food sources and/or snow cover [14,15]. They are often particularly sensitive to increased average and/or upper temperatures; thus, climate change can lead to decreased reproduction and overwinter survival, as individuals reduce their foraging time to avoid overheating [13,16]. Reduced snowpack may result in montane mammals losing their buffer against low temperatures, and could lead to exhaustion of fat reserves and ultimately overwinter starvation [17], especially for hibernators [18]. Changes in precipitation, especially increased flooding and rain on snow events, can increase mortality for montane mammals that burrow in meadows. Finally, severe weather in the already extreme environment of montane ecosystems can lead to death, particularly in winter [19–20].

We used a detailed dataset to examine fine-scale range shifts in a species' distribution to understand how recent global change is affecting a montane species at the trailing edge of its range and whether refugia are reducing the effect. Specifically, we tested whether changes in temperature or precipitation explained range dynamics of a hibernating semi-fossorial montane sciurid, the Belding's ground squirrel (*Urocitellus beldingi*). Sites in the Sierra Nevada originally surveyed by Joseph Grinnell and colleagues primarily in the first part of the twentieth century were resurveyed 45–101 years later. Montane ecosystems in the western US have already experienced multiple direct and indirect effects of climate change, including an increase in average and extreme temperatures, precipitation falling as rain versus snow, severe wildfires and earlier snowmelts [21–24]. Many Sierra Nevadan bird, butterfly, mammal and tree species have shifted their ranges upslope over the last 35–100 years, although some have shifted downslope [25–28].

For our study, we predicted that climate change will have led to local extirpations of *U. beldingi* outnumbering colonizations at the lower latitude and lower elevations of its range [1,26,29], and that extirpations would be more likely at sites experiencing greater increases in temperature and/or changes in precipitation. We also examined whether a human-mediated increase in food and water availability promoted persistence and increased abundance at such ‘anthropogenic refugia’, particularly at the trailing edge. Finally, we used historically validated species distribution models to project the range of *U. beldingi* to 2080 and to predict impacts of an increasingly warm and wet future. Our findings reveal the surprising ameliorating effect of anthropogenic refugia on the severe climate-related decline of a montane mammal at its trailing edge.

## 2. Material and methods

### (a) Surveys

**Figure 1.**
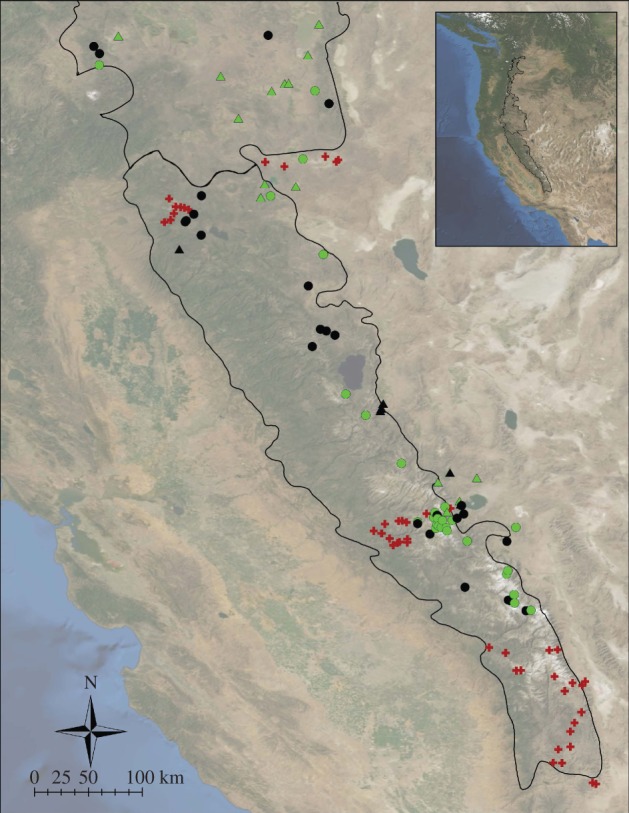
Surveyed sites in California showing where *U. beldingi* has persisted in green (*n* = 43) and been extirpated in black (*n* = 31). Triangles indicate anthropogenic sites (e.g. campgrounds or agricultural fields). Red crosses (*n* = 47) mark resurvey sites where *U. beldingi* were absent in the historical and modern time periods; there are no known instances of colonization.

The Belding's ground squirrel, *U. beldingi*, is a 250 g social, ground-dwelling mammal feeding primarily on grasses and commonly found in meadows in mid- to high-elevations in western North America [30]. Historical occurrences of *U. beldingi* from 1902 to 1966 were collected from detailed field notes from the Museum of Vertebrate Zoology (MVZ) at the University of California at Berkeley (http://bscit.berkeley.edu/mvz/volumes.html). We deemed this time period as historical because it predates the manifestation of the majority of effects of anthropogenic climate change in California [31]. We found locations and survey information for 74 sites where *U. beldingi* were historically observed and/or trapped (figure 1). Resurveys in or near the Sierra Nevada and southern Cascades were conducted in July and August of 2010 and 2011 within a mean of 13 (s.d. = 8) days of the original survey date. Surveys in northern California (Modoc and Siskiyou counties), where *U. beldingi* enter into and arouse from hibernation earlier in the year, were conducted in May. One to four observers walked each site for an average of 96 min during an average of 2.6 visits per site within one week, while visually scanning for *U. beldingi* and listening for alarm calls commonly used by *U. beldingi* when they are disturbed or spot a predator. Wherever possible, short-grass areas (meadows, fields, parks and campgrounds) within a 2 km radius of the original site were surveyed as well. If *U. beldingi* were seen within 2 km of the historical survey point, the site was considered occupied. Two kilometres is a conservative definition of a site, considering that *U. beldingi* normally disperse less than 500 m from their natal territory [32], and is similar to the scale used by others [25].

As local population extirpations could merely be offset by colonizations at other sites on the landscape as part of metapopulation dynamics, we determined whether any sites were now occupied where *U. beldingi* were not recorded during historical surveys. In total, we resurveyed 47 additional sites where *U. beldingi* were not observed or trapped during historical trapping surveys, excluding sites that were lower than the lowest elevation where we observed *U. beldingi*. Data for these surveys were by the MVZ Grinnell Resurvey Project (GRP), a research project focused on understanding changes in California fauna over the past century [25,26].

### (b) Analysis of persistence and abundance

We analysed detectability from resurveys at 74 sites that were historically occupied by *U. beldingi* through occupancy modelling using the program PRESENCE [33,34]. We used a single-season model without covariates. Detection probability was estimated per visit (*p*) and per site (*p_x_*) over the total number of visits (*v*) as: *p_x_* = 1−(1−*p*)*^v^*.

Factors determining persistence or extirpation at resurvey sites were analysed using classification tree analysis (Random Forests package, [35]) in R v. 2.13.1 [36]. First, we used the ‘cforest’ function from package ‘party’ [37], including all variables with 10 000 iterations, to obtain unbiased conditional variable importance (‘varimp’) estimates (conditional = TRUE, nperm = 100). We then ran all possible combinations of the 10 factors with the highest variable importance estimates (table 1) that were uncorrelated (*r* < 0.5) using the ‘randomForests’ package [38] to identify the model with the lowest error rate. Finally, we used the conditional tree function in R package ‘party’ (‘ctree’, developed by [39]), allowing the tree to show splits up to a significance level of *p* = 0.05, to corroborate variable selection.

**Table 1.** Factors tested and their predicted effect on persistence of *U. beldingi*. Relative importance values are from the varimp analysis, which was one method used to identify the model that best predicted the observed patterns of extirpation. Negative values indicate that including the factor makes the model worse.

factor	predicted effect on persistence	relative importance
modern temperature of coldest quarter	decrease	0.023
historical temperature of coldest quarter	decrease	0.023
change in precipitation of driest quarter	decrease	0.012
human modification^a^	increase	0.0096
historical precipitation of wettest quarter	decrease	0.0093
modern temperature of warmest quarter	decrease	0.0085
historical temperature of warmest quarter	decrease	0.0072
modern precipitation of wettest quarter	decrease	0.0055
change in temperature of warmest quarter	decrease	0.0044
historical precipitation of driest quarter	decrease	0.0022
change in temperature of coldest quarter	decrease	0.00025
change in precipitation of wettest quarter	decrease	−0.000062
modern precipitation of driest quarter	decrease	−0.000087
meadow area within 500 m^2^ (m^2^)	increase	−0.00019
private jurisdiction	decrease	−0.000397
distance to running water (m)	decrease	−0.00041
focal meadow area (m^3^)	increase	−0.00071
distance to water body (m)	decrease	−0.00071

^a^Human-mediated increase in food and/or water availability.

Climate data were obtained from the parameter elevation regressions on independent slopes model (PRISM, [40]) at 800 m resolution. PRISM is an expert-tuned meteorological interpolation system that bases its predictions on observed weather measurements. The set of climate variables initially examined were calculated for: (i) BIOCLIM variables [41] of climate data for 1900–1939, covering the dates of 60 of the 74 historical surveys; (ii) similar measures from an equivalently long modern period from 1970–2009; and (iii) the change between the two time eras. We hypothesized that temperature and precipitation extremes influenced the pattern of persistence and extirpation; so we considered average temperature of warmest quarter (BIO10), average temperature of coldest quarter (BIO11), average precipitation of wettest quarter (BIO16) and average precipitation of driest quarter (BIO17). None of these factors had pairwise correlations with |*r*| ≥ 0.5, which is a minimal correlation for detecting colinearity [42].

We also examined persistence at a site as a function of land ownership (federal, state or city jurisdiction versus private), human modification (relatively unmodified versus supplemented with food through high human activity (such as campgrounds) or with water (such as county parks), or both (such as agricultural fields)), and several landscape features that were calculated using GIS (ESRI ArcMap v. 10.0). The latter included the size of the focal meadow area, total meadow area (area of meadow habitat within a 500 m radius of the survey site), distance to nearest running water (spring, creek, or river) and distance to nearest water body (lake or pond). Table 1 lists these factors and their predicted relationship with persistence (also see electronic supplementary material, table S1). We predicted that private jurisdiction would have a negative effect on persistence due to increased habitat conversion and hunting (*U. beldingi* are considered agricultural pests in some places, [43]). We predicted that human modification, in the form of human-mediated increase in food and water availability, would show a positive correlation with persistence. Finally, the earlier-mentioned landscape variables should be positively correlated with suitable *U. beldingi* habitat and thus with *U. beldingi* persistence.

We conducted two tests to examine further the effect of human-mediated increase in food and water availability on *U. beldingi* persistence. First, we used a chi-squared analysis to test whether human habitat modification provided climate refugia by increasing persistence at low elevations along the edge of the range, focusing on the lowest 20 per cent of sites (*n* = 15, elevation range: 1286–1551 m). Second, to determine whether *U. beldingi* densities differed between human-modified and unmodified sites, we calculated the trap success rate (unique animals trapped/(number of traps/number of trap periods)) for sites where animals were successfully captured in 2010 at eight sites (six of the resurvey sites described earlier and two additional non-historical sites that were opportunistically surveyed). Sites were evenly apportioned between human-modified and natural meadows (average elevation of unmodified sites = 2545 m; average elevation of human-modified sites = 2447 m). Each site was trapped over multiple morning and evening sessions lasting more than 90 min using 10–41 baited Tomahawk live traps (16″ × 5″ × 5″; Tomahawk, Hazelwurst, USA) per trap session. Trapped animals were earpunched and released at the site of capture. We used a Welch two-sample unpaired *t*-test in R to determine whether human-modified sites were more likely to support a higher density of *U. beldingi* than natural meadows.

### (c) Species distribution modelling

Generalized additive models (GAMs, [44]) were used to model species' distributions using custom code and the ‘mgcv’, ‘dismo’ and ‘raster’ packages in R. We used a separate dataset of presence and absence (non-detection) points (see the electronic supplementary material, figure S1) gathered primarily from GRP surveys or downloaded from MANIS (Mammal Networked Information System, www.manisnet.org) in September 2011 to train the models, thinning out sites found within 1 km of each other. In total, we analysed 52 historical and 91 modern presence sites in California, 50 historical and 152 modern absence sites in California, and 58 historical and 17 modern presence sites in the western US outside of California. No non-California absences were analysed because we were unable to estimate detectability at these sites.

The performance of these models was assessed using the presence/extinction data with the area under the receiver operator curve (AUC). AUC indicates how reliably a model can rank environmental suitability of presences relative to absences, and spans from 0 to 1, with values greater than 0.5 indicating performance better than random [45]. Model output was thresholded to delineate presences from non-presences on prediction maps at the value that minimized the differences between the proportion of presences and absences/extirpations correctly predicted. Projection maps were partitioned at the prevalence of the training data (proportion of presences to total sites) to delineate areas where the species was predicted to persist or be extirpated. Using variables selected in the tree model as a basis, we chose average temperature of coldest quarter and average precipitation of wettest quarter for each time period. We then used future values of each variable to project forward to average conditions from 2065 to 2095 (hereafter called ‘2080’) modelled under the CCCMA and Hadley general circulation models [46] for the A2 and B2 emissions scenarios (highest and lowest emissions scenarios, respectively).

For the contiguous US west of −103.772°, CCCMA A2 for 2080 projects a change of +3.92°C (42% increase) in average annual temperature and  +4.83 cm (9% increase) in average annual precipitation, whereas Hadley A2 projections for the same time period are +4.03°C (43% increase) for average annual temperature but −5.36 cm (10% decrease) for average annual precipitation. All data were projected to the California area of the Sierra Nevada and Eastern Cascades Slopes and Foothills US Environmental Protection Agency Ecoregions, where 61 of 74 of the resurvey sites occur; 12 of the remaining 13 extant sites are within 10 km of these regions' boundaries (see the electronic supplementary material, figure S1).

## 3. Results

### (a) Survey results

Detectability of *U. beldingi* per visit averaged 0.94 (95% CI: 0.91–0.97) and per site averaged 0.96 (95% CI: 0.94–0.99), yielding a low probability of false absence at surveyed sites (mean = 0.04). Thus, we considered *U. beldingi* ‘extirpated’ at sites that were occupied historically but where we had no detections during resurveys.

*Urocitellus beldingi* were present at only 43 of the 74 (58.1%) sites in California where it had been observed or trapped between 1902 and 1966 and present at none of the 47 historically unoccupied sites (figure 1). Thus, we found a 42 per cent rate of extirpation and 0 per cent rate of colonization for *U. beldingi* in its historically surveyed range.

### (b) Persistence and abundance related to climate and human modification

**Figure 2.**
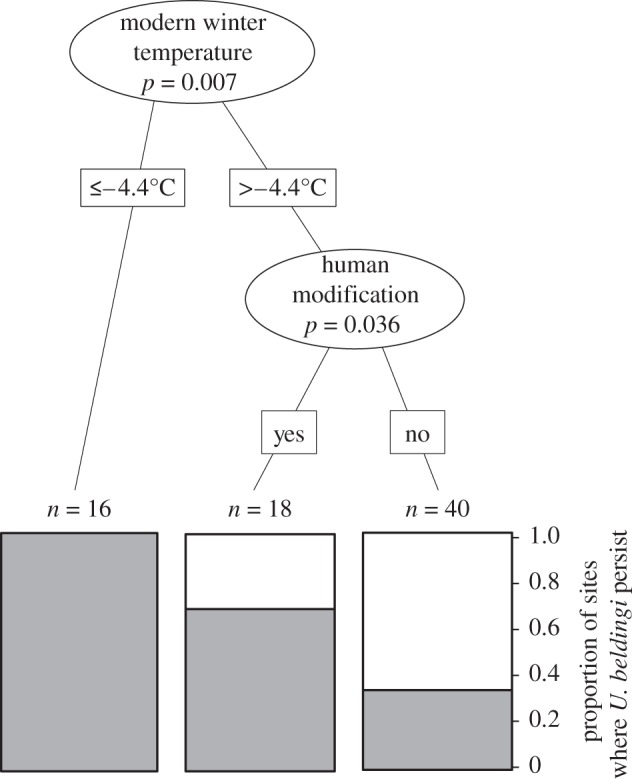
Classification tree showing the effect of climate factors and human modification on *U. beldingi* persistence. Grey shading denotes the proportion of persistent sites modelled in each category. Increased probability of persistence is associated with low (average less than or equal to 4.4°C) coldest quarter temperatures and ‘anthropogenic refugia’ created by a human-mediated increase in food and water availability. Although modern wettest quarter precipitation and change in warmest quarter temperature were included in the best model, they were not significant at *p* < 0.05 and thus do not appear in the figure.

Classification tree analysis found the greatest support for a model of persistence that included human modification (positively related), modern average coldest quarter temperature (negatively related), change in average warmest quarter temperature (negatively related) and historical average wettest quarter precipitation (negatively related), although the latter two factors were not significant at the level of *p* < 0.05 and thus do not appear in the final classification tree (figure 2). The final classification tree specifically indicates that average coldest quarter temperature of −4.4°C is a predictor for *U. beldingi* persistence; below this temperature, no sites were extirpated. Classification error rate (out-of-bag estimate) was 18.92 per cent, indicating that the overall percentage of cases correctly classified was nearly 80 per cent. Specificity (% of extirpated sites correctly classified) was 84 per cent; sensitivity (% of persistent sites correctly classified) was slightly lower at 79 per cent. As predicted, the probability of site extirpation was positively related to increases in average coldest- and warmest-quarter temperatures and wettest-quarter precipitation (figure 2).

**Figure 3.**
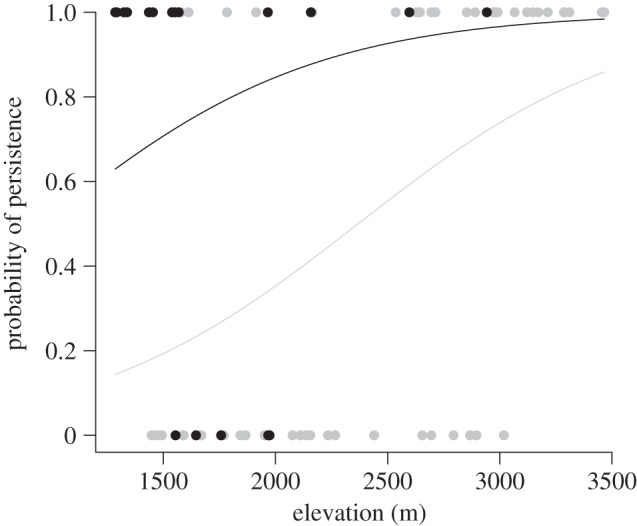
Probability of *U. beldingi* persistence increases with elevation and is lower in natural sites (grey dots and line) than in human-modified sites (black dots and line). Such ‘anthropogenic refugia’ are especially important at low elevations (trailing edge).

Human modifications through food or water supplementation (e.g. a campground or an agricultural field) enhanced persistence of *U. beldingi* (figure 2). In addition, persistence at low elevation sites, which were also warmer (see the electronic supplementary material, figure S2), was greater when sites were associated with human modification (null deviance reduction = 16.5; figure 3). Although the historical and modern elevation ranges were identical (historical average = 2368 m, modern average = 2227 m, range = 1286–3465 m), the range of sites where *U. beldingi* persisted would have retracted upslope by 255 m if human-modified sites were excluded (unmodified modern average = 2653 m, range = 1541–3465 m). Human modification also resulted in higher *U. beldingi* abundance. Trap success at four natural meadow sites (X ± s.d. = 0.084 ± 0.074 animals/trap period) was significantly less (*t* = 2.9, d.f. = 6, *p* = 0.026) than at the four human-modified sites (0.28 ± 0.094).

### (c) Projections of the species' range to the present and the future

**Figure 4.**
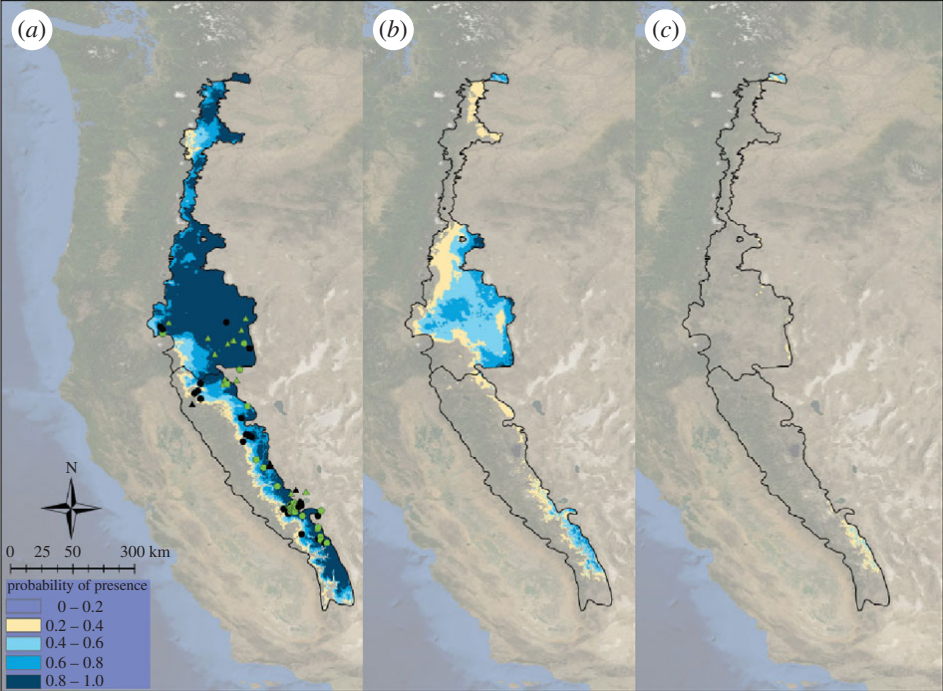
*Urocitellus beldingi* species distribution model (GAM) results projecting (*a*) current distribution from historical presences and absences, (*b*) future distribution from 2080 Hadley, A2 scenario and (*c*) future distribution from 2080 CCCMA, A2 scenario. Probability of persistence is marked by quantile (0–0.2 not shown, 0.2–0.4 in beige, 0.4–0.6 in light blue, 0.6–0.8 in blue and 0.8–1 in dark blue).

Historical presence locations across the western US gathered from museum data (see the electronic supplementary material, figure S1) were used to train the GAM, which was then used to project the distribution of *U. beldingi* from the past to the present (figure 4*a*). This projection accurately predicted persistence and absence (i.e. extirpation) of the species from the historical to the modern time period (AUC = 0.76), with 70–71% of presence and absence sites correctly projected, respectively.

Projections of *U. beldingi* from species distribution models indicate that climate change will cause large-scale extirpations that will further contract the already-reduced range of this species (figure 4*b,c*). Currently occupied suitable habitat represents only 58 per cent of the historically occupied habitat. Projecting to the future suggests a loss of between 72 per cent (Hadley A2) and 99 per cent (CCCMA A2) of historically suitable habitat within California.

## 4. Discussion

Our study found a remarkable rate of extirpations (42%) without any concurrent colonizations in the previously surveyed range of a montane mammal. The disappearance of *U. beldingi* from much of its known California range in less than a century is alarming, as it is an important prey species for raptors and carnivores and a potential indicator of meadow habitat. Nevertheless, the limits of *U. beldingi*'s elevation range have not shifted because human modification of habitats at low elevations has provided anthropogenic refugia, a result that urges further study to understand the mechanism of this effect.

### (a) Signatures of climate change on *Urocitellus beldingi* extirpations

Higher temperatures in the coldest and warmest quarter were negatively related to the persistence of *U. beldingi* along the trailing edge of its range, with a higher rate of extirpations at lower elevation sites. Climate change may have exceeded a critical temperature threshold, either acutely or cumulatively, or affected persistence indirectly by preventing feeding or other activities. Species at mid-elevations in the mountains of central and northern California live close to the freezing line, where temperature increases are having detectable effects on snowcover, potentially resulting in negative impacts on the physiology and phenology of montane species [47,48].

A scenario of freezing due to loss of winter snowcover was proposed to explain extirpations seen in another montane mammal population, the American pika (*Ochotona princeps*) in the Great Basin [17]. Nevertheless, pika in the Sierra Nevada and the southern Rocky Mountains appear to be persisting [49,50]. *Urocitellus beldingi* may show a stronger response to climate change in California because the talus habitat of *O. princeps* in California is more thermally protective and more ubiquitous with little elevational limit [49], whereas the meadow habitat of *U. beldingi* is limited elevationally, providing *U. beldingi* less opportunity to colonize new habitats as climate changes. Moreover, shifting temperature regimes and seasonality may be causing a mismatch with *U. beldingi* hibernation behaviour, as some ground squirrels are known to use abiotic and biotic cues to determine the start and end of hibernation, respectively [18,51]. A study of range changes over the past 80 years in the Ruby Mountains of Nevada found a similar tendency for *U. beldingi* to disappear from low- and mid-elevation sites [52], although the change over time was not statistically significant.

Interestingly, another montane hibernating mammal of western North America, the yellow-bellied marmot (*Marmota flaviventris*), appears to be thriving in the interior of its range, both in terms of average body size increases and population growth, due to an earlier green-up leading to a shorter hibernation time [53]. Marmots may be able to tolerate climate change in a way that *U. beldingi* cannot because marmots are habitat and dietary generalists. Moreover, *U. beldingi*'s meadow habitat may be changing in response to shifting temperature and precipitation regimes. Woody species increase and compete with the grasses that they consume as meadows dry out [54]. However, *U. beldingi* has increased in body size over the past century [55], indicating that the species is not suffering from a lack of food resources where it persists.

Wettest quarter precipitation was negatively correlated with persistence in *U. beldingi*. The adverse effect of increased precipitation may be due to direct mortality or indirect metabolic stress [56,57]. Simply being wet causes terrestrial mammals to expend more energy, up to 100 per cent above basal metabolic rate [58]. Heavy spring snowstorms have decimated Belding's ground squirrel populations [19]. Changes in precipitation that lead to increased flooding and rain on snow events can reduce survival for montane mammals, especially burrowing meadow species. Moreover, extreme winter precipitation events are projected to become more common in the western United States [23].

We were not able to test whether the large range reduction we documented for *U. beldingi* occurred directly from the effects of increased temperature and precipitation or instead indirectly resulted from an ecological impact caused by climate change. For example, a disease such as plague, which can decimate entire populations of *U. beldingi* [59], may have increased owing to warmer or wetter climates. There may also be non-climate causes. A direct mechanistic modelling of physiological limits [60] might help to disentangle the direct effects of climate change from other causes.

### (b) Anthropogenic refugia buffer climate change

The absolute elevation limits of the *U. beldingi* California range have not changed over the past century, despite their disappearance from more than 40 per cent of surveyed sites and the strong negative correlations between elevation and extirpation. Anthropogenic refugia appear to explain this discrepancy. The elevational range of *U. beldingi* would have retracted upslope by 255 m if sites with human-mediated increases in food and water availability were excluded. Human modification also had a positive effect on abundance. Likewise, occupancy was less affected by climate change in the Sierra Nevada for avian species that frequently use human-modified habitats [26].

Our study demonstrates a potential protective effect of anthropogenic refugia for montane species. Food and/or water supplementation, or other aspects of human presence, could modify the effects of warming and shifting precipitation regimes. Although we found that specific human modification had a positive effect, activities that convert meadow habitat into more urban or suburban uses would likely not support *U. beldingi* populations. Identifying both artificial and natural refugia that are buffered from climate change may allow the persistence of vulnerable species in the face of future climate change.

### (c) Future projections of *Urocitellus beldingi* distribution

We projected that the range of *U. beldingi* along its trailing edge would decrease severely in a warmer future using historically validated models. Two projections based on greenhouse gas emissions within the scope of current activity (i.e. A2 scenario) showed greater than 50 per cent range retraction of preferred habitats for *U. beldingi* in California from current habitat suitability, which already has been reduced by 42 per cent compared with a century ago. The wetter future of CCCMA A2 projected the greatest decline, with up to 99 per cent loss of a suitable habitat. Although these projections only consider climate and are probably not reliable numeric predictions of future *U. beldingi* habitat [61,62], they corroborate our other results that indicate that the species is being, and will continue to be, negatively impacted by climate change along the warmer, and potentially also the wetter, trailing edge of its distribution.

## 5. Conclusions

Long-term occupancy data are needed to examine how species' ranges have shifted as a result of recent changes in climate and land-cover. This study provides a rare glimpse of the effect of a century of climate and land-use change along the trailing edge of the range of a montane mammal that was facilitated by the detailed historical field notes of Joseph Grinnell and colleagues. Our study shows an extreme population reduction related to warm and wet climate, with a 255 m upslope retraction mediated only by anthropogenic refugia, and points to the need to understand the exceptions as well as the patterns of shifts in the trailing edge of species ranges.
